# COVID-19 vaccination during pregnancy: a systematic review and meta-analysis

**DOI:** 10.1186/s12884-023-05374-2

**Published:** 2023-01-20

**Authors:** Arman Shafiee, Omid Kohandel Gargari, Mohammad Mobin Teymouri Athar, Haniyeh Fathi, Marjan Ghaemi, Sayed-Hamidreza Mozhgani

**Affiliations:** 1 School of Medicine, Alborz University of Medical Sciences, Karaj, Iran; 2 Student Research Committee, Alborz University of Medical Sciences, Karaj, Iran; 3 School of Medicine, Shahid Beheshti University of Medical Sciences, Tehran, Iran; 4 Vali-E-Asr Reproductive Health Research Center, Family Health Research Institute, Tehran University of Medical Sciences, Tehran, Iran; 5 Department of Microbiology, School of Medicine, Alborz University of Medical Sciences, Karaj, Iran; 6 Non-Communicable Disease Research Center, Alborz University of Medical Sciences, Karaj, Iran

**Keywords:** COVID-19, Vaccination, Pregnancy

## Abstract

**Background:**

SARS-CoV-2 exposure during pregnancy is related to adverse effects for both the mother and the infant. SARS-CoV-2 vaccination has lowered the risk of symptomatic disease substantially. Recently published studies have evaluated the outcomes of women who received the COVID-19 vaccine during pregnancy; systematic evidence regarding vaccination safety is crucial to ensure that COVID-19 vaccination is not associated with adverse pregnancy and neonatal outcomes.

**Methods:**

Pubmed/MEDLINE, EMBASE, Scopus, Web of Science, and Clinicaltrials.gov were searched from each database's inception through April 7, 2022. All interventional and observational studies comparing neonatal or pregnancy outcomes between pregnant women who received COVID-19 vaccines during their pregnancy and unvaccinated pregnant women were included. The random-effects model was used in the meta-analyses.

**Results:**

A total of 11 studies comprising 756,098 pregnant mothers were included. The rate of neonates with 5-min Apgar score ≤ 7 (log RR -0.08 (95% CI: -0.15 to -0.00), (*P* = 0.03)) and pregnant mothers with preterm birth (log RR -0.11 (95% CI: -0.21 to -0.01), (*P* = 0.02)) was significantly lower among vaccinated group. No significant difference was observed in adverse neonatal outcomes (log RR -0.07 (95% CI: -0.17 to 0.03)), small for gestational age (log RR -0.06 (95% CI: -0.14 to 0.02)), caesarean delivery (log RR 0.05 (95% CI: -0.05 to 0.15)), postpartum hemorrhage (log RR -0.05 (95% CI: -0.13 to 0.02)), stillbirth (log RR -0.05 (95% CI: -0.54 to 0.45)).

**Conclusions and relevance:**

In this systematic review and meta-analysis, no evident differences were observed when comparing vaccinated pregnant mothers with those who had not received COVID-19 vaccines. Based on low certainty of evidence, vaccination during pregnancy was accompanied by a favorable Apgar score in neonates and fewer preterm births.

**Supplementary Information:**

The online version contains supplementary material available at 10.1186/s12884-023-05374-2.

## Background

The world has been struggling with the Coronavirus disease 2019 (COVID-19) pandemic for two and a half years. As of September 21, 2022, there have been more than 610 million cases confirmed, and more than 6.5 million people have lost their lives due to this pandemic [1]. There have been concerns about how COVID-19 could affect pregnant mothers and their neonates. Studies revealed that COVID-19 in pregnant women is accompanied by more severe manifestations compared with non-pregnants [2, 3]. It was also suggested that pregnant women with COVID-19 are at higher risk for preterm birth, preeclampsia, eclampsia, stillbirth, neonatal morbidity, and mortality than pregnant women without COVID-19 [4, 5].

Till now, vaccines are the most reliable option for controlling the severity of this disease [6]. The World Health Organization (WHO) has approved the emergency use of several vaccines, including AstraZeneca/Oxford, Johnson and Johnson, Moderna, Pfizer/BioNTech, Sinopharm, Sinovac, COVAXIN, and Nuvaxoid [7]. Most severe COVID-19 cases in pregnant women were reported from unvaccinated patients [8]. The American College of Obstetricians and Gynecologists (ACOG) recommends the vaccination of pregnant women with one of the mRNA vaccines in the U.S. and states that these vaccines are preferred to the J&J vaccine, whereas WHO recommends vaccination of pregnant women with the Sinopharm vaccine when the benefits outweigh the possible risks [9, 10].

Little data is available regarding the safety of these vaccines during pregnancy in the concept of randomized controlled trials [11, 12]. Although some studies have proven the safety and efficacy of vaccines in pregnant women [13–15], there is still a lack of data, and vaccine hesitancy is present among pregnant women [16]. Evidence from systematic reviews and meta-analyses is desperately needed on this topic, and one of the factors affecting the acceptance of vaccination is the certainty of systems to assess vaccine safety [16]; therefore, in this study, we aimed to evaluate the current evidence regarding the safety of vaccination and its possible effect on pregnancy and neonatal outcomes among vaccinated pregnant women compared with unvaccinated pregnant women.

## Methods

### Search strategy

A systematic review and meta-analysis comparing the possible effect of COVID-19 vaccination on neonatal and pregnancy outcomes was conducted in accordance with Cochrane collaboration procedures [17]. The Preferred Reporting Items for Systematic Reviews and Meta-Analyses (PRISMA) was used in this study [18]. The protocol of this study is registered at PROSPERO under the number CRD42022323965.

Pubmed/MEDLINE, EMBASE, Scopus, Web of Science, and Clinicaltrials.gov were searched by our reviewers (A.S and O.K and H.R). The following terms with their combinations were searched: SARS-CoV-2, COVID-19, Vaccines, and Pregnanant (Full search strategy is provided in the Supplementary Table 1). All publications published up to April 7, 2022 were retrieved. Additionally, in order to find relevant studies, we hand-searched the reference part of the relevant studies.

### Study selection and data extraction

Studies were included based on the following PICOT criteria: 1) Population: adult pregnant women; 2) Exposure: received at least one dose of COVID-19 vaccines (in any types) during their pregnancy; 3) Comparator: unvaccinated pregnant women; 4) Outcome: studies evaluating relative outcomes in both vaccinated and unvaccinated group; and 5) Type of study: all types of original articles were applicabale. We included studies published in English language with accessible full text. Additionally, studies that reported the outcome only in vaccinated group were excluded. The titles and abstracts of the studies were reviewed by three independent reviewers (A.S and O.K and H.R), followed by full text review. An Excel spreadsheet was designed to include the Data extracted from text, tables, figures, graphs, and supplementary materials. Two reviewers (O.K and H.R) independently extracted the following data: author, year of publication, Journal/full paper or abstract, country, population, study type,, number of included patients in the study, type of vaccine, as well as relevant outcome data. To reach an agreement, discepancies were resolved through discussion with a third reviewer (M.T).

### Quality assessment

Two reviewers (O.K and H.R) independently assessed the included studies using the National Heart, Lung, and Blood Institute (NHLBI) risk of bias checklist [19]. considering the 14 questions designed to assess the quality of observational cohort and cross-sectional studies,studies with 10 or more yeses are rated as “Good”, 7–9 yeses as “Fair”, and fewer than 7 yeses are rated as “Poor” [20]. We used the Grading of Recommendations, Assessment, Development and Evaluations (GRADE) framework and GRADEpro GDT to evaluate the certainty of evidence for our outcomes [21].

### Outcomes

Neonatal outcomes evaluated in this meta-analysis were: 1) adverse neonatal outcomes (ANO), which was defined as neonatal respiratory complications and Neonatal intensive care unit (NICU) admission; 2) 5-min Apgar score ≤ 7, which was defined as the assessment of 5 domains (skin color, heart rate, reflexes, muscle tone, and respiration) in neonates immediately after birth; and 3) small for gestational age (SGA). Pregnancy outcomes were as follow: 4) rate of caesarean delivery; 5) rate of post-partum haemorrhage (PPH); 6) preterm birth defined as gestational age < 37 weeks at delivery; and 7) stillbirth.

### Data synthesis and analysis

Since the indicators differed among studies, pooling of our data was carried out using the Restricted-maximum-likelihood random-effects model. A log risk ratio (log RR) was calculated to sum up the overall effects of outcomes. Furthermore, to present the results in forest plots, the log RR was back-transformed to RR for ease of interpretation. A *p*-value of < 0.05 was considered as the threshold for significance of the effect estimate. We assessed the heterogeneity of the studies using Cochrane Q-test for heterogeneity (cut off point set as < 0.1 showing significant heterogenity) and I^2^ statistic. Studies were classified into three groups of low, moderate, and high level of heterogeneity based on the respective value of I^2^ < 50%, 50% to 75%, and > 75%. As the most commonly used variable for measurement of heterogeneity, the I^2^ value is in direct relationship with the number of included trials, making the comparison of I^2^ values between analyses challenging. Therefore, both I^2^ and Tau values for each analysis were reported in our study. Publication bias was appraised using funnel plots inspection and Egger's regression test for funnel plot asymmetry for outcomes. At least ten studies must be included based on the Cochrane handbook's suggestions in order to assess publication bias [9]. To evaluate the effect of individual studies on the pooled results, we performed a leave-one-out sensitivity analysis. The analysis was carried out using R (version 4.1.3) (R Core Team, 2020), the metafor package (version 3.0.2) (Viechtbauer, 2010), and the meta package.

## Results

**Fig. 1:**
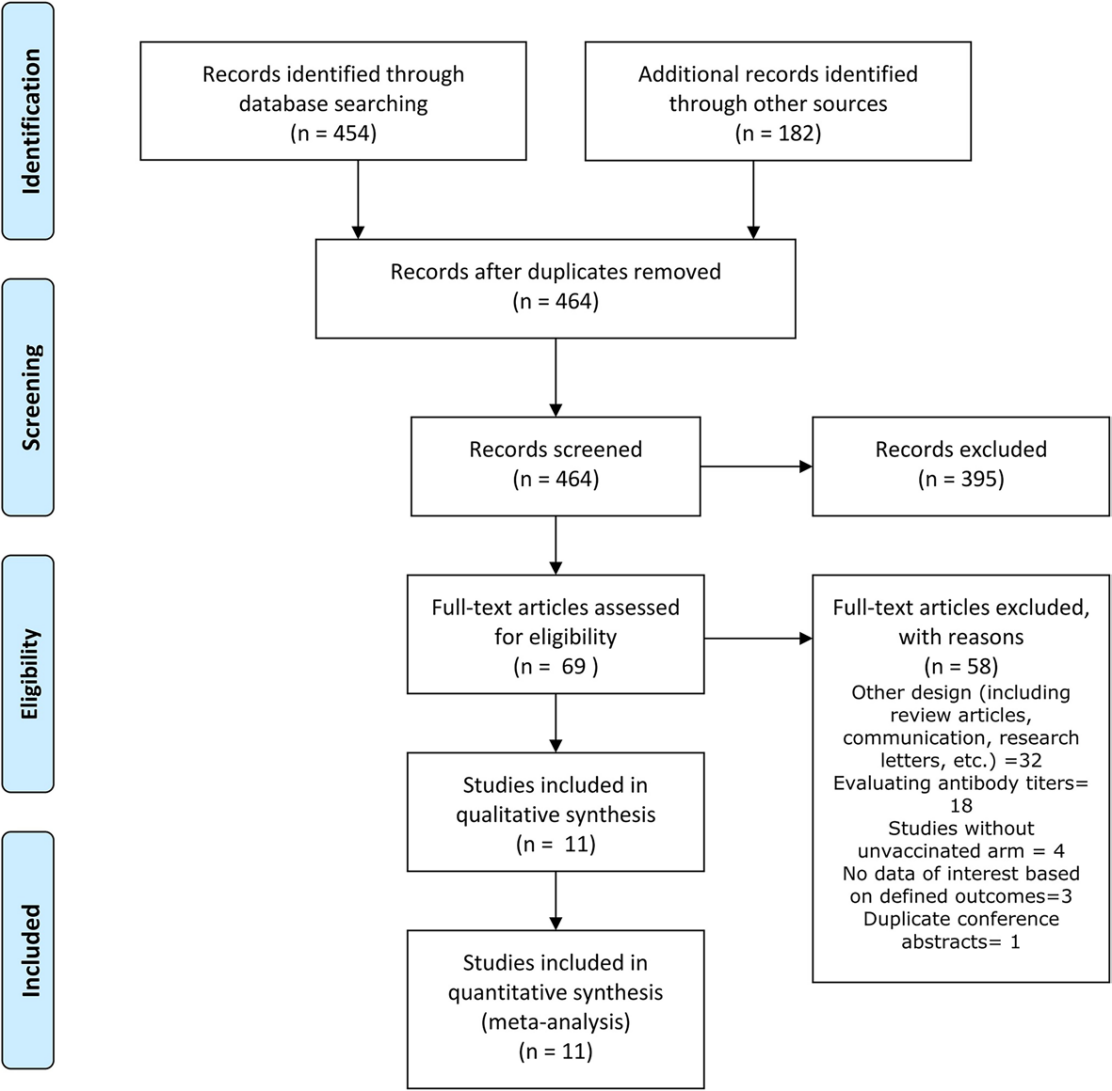
Study Flowchart

Based on our initial search, we identified 636 studies, removed 172 duplicates, and after the titles and abstracts screening, we reviewed full-texts of 62 articles, and eventually included 11 studies. (Fig. 1).

### Characteristics of the included studies

Three studies were conducted in European countries, including Sweden & Norway (*n* = 1), England (*n* = 1), and Romania (*n* = 1); two of the studies were conducted in America, including the United States (*n* = 1) and Canada (*n* = 1). There were six studies conducted in Asia, which were all from Israel. mRNA vaccines (including BNT162b2 (Pfizer-BioNTech) and mRNA-1273 (Moderna)) were the most common vaccines for which the results regarding their effects have been provided among the included studies. Detailed characteristics of each study are provided in Table 1.

**Table 1:** Baseline characteristic of included studies

**Authort/Year**	**Country**	**Journal/full paper or abstract**	**Type of study**	**Study duration**	**Total Patients** **(n)**	**Age**	**Type of Vaccine**	**Evaluated Outcomes**	**Quality**
Wainstock, T. [22] / 2021	Israel	Vaccine	Retrospective cohort	January-June 2021	4399	Vaccinated: 30.6 ± 5.3/Unvaccinated: 28.2 ± 5.7	Pfizer-BioNTech	Pregnancy related hypertensive disorders Oligohydramnios Polyhydramnios Pathological presentation Meconium stained amniotic fluid Gestational age at delivery (mean ± SD) Apgar < 7 at 5 min Non reassuring fetal monitoring Cesarean delivery Vacuum delivery Placental abruption Postpartum hemorrhage Maternal postpartum fever Length of maternal hospitalization, days (median, range) Following cesarean delivery, Following vaginal delivery Birthweight, gr. (mean ± SD) Small for gestational age Newborn respiratory complications Newborn fever Length of newborn hospitalization, days (median, range) Following cesarean delivery,Following vaginal delivery	Good
Theiler, R. N [23]/ 2021	United States	American journal of obstetrics and gynecology	Cohort	December2020—April 2021	2002	30.1 ± 5.2	Johnson & Johnson/ Moderna/Pfizer-BioNTech"	Length of stay Quantitative blood loss > 1000 mL Transfusion Thromboembolism Stroke Eclampsia or preeclampsia up to 72 h from delivery Gestational hypertension Low birthweight (< 2500 g) Very low birthweight (< 1500 g) Stillbirth Spontaneous vaginal Operative vaginal Cesarean delivery Gestational age at delivery Maternal death during hospitalization Intrapartum neonatal death within 7 d of birth, ≥ 2500 g, ≥ 37 wk, Hypoxic-ischemic encephalopathy, Uterine rupture Unplanned maternal ICU admission, Birth trauma, Return to the operating room, Neonatal ICU admission within 1 d of birth, 5-min Apgar score of < 7, Postpartum hemorrhage with transfusion Third- or fourth-degree laceration	Good
Rottenstreich, M. [24]/ 2022	Israel	Bjog	Retrospective cohort	January-April 2021	5745	vaccinated:30.6 ± 5.8 /unvaccinated:29.5 ± 6	Pfizer– BioNTech	Birthweight > 4000 g, Birthweight, g, LGA, SGA, Male gender, 1-min Apgar score ≤ 7, 5-min Apgar score ≤ 7, Intrauterine fetal death, NICU admission, Meconium aspiration syndrome, Jaundice, TTN, Mechanical ventilation, Seizures, Hypoglycemia, Sepsis, Encephalopathy Intracranial hemorrhage, Birth asphyxia, Composite adverse neonatal outcome, Gestational age at delivery, Gestational age at delivery < 34 weeks, Gestational age at delivery < 37 weeks, Induction of labor, Oxytocin augmentation of labor, Epidural analgesia, Meconium-stained amniotic fluid, Chorioamnionitis, Caesarean delivery, Elective caesarean delivery, In-labor caesarean delivery, Home/car delivery, Vacuum-assisted delivery, Hospitalization length, days Prolonged hospital stays, Episiotomy, Maternal ICU admission, Postpartum hemorrhage, Placental abruption, Hemoglobin drop, g/dl, Hemoglobin drop > 4 g/dl, Puerperal fever, Blood products transfusion, Composite adverse maternal outcome	Good
Mayo [25]/ 2021	Israel	medrxiv	Cohort	January-June 2021	1702	Control group:29.5 ± 5.5/Past SARS-Cov-2 group:28.7 ± 5.5/Vaccinated group:31.4 ± 6.1	Pfizer– BioNTech	Infant sex Birthweight NICU Maternal comorbidities, Hypertensive disorders Diabetes or gestational diabetes Gestational age, weeks Preterm delivery (< 37)	Good
Magnus [4] /2022	Sweden & Norway	JAMA	Registry-based retrospective cohort	January 2021—January 2022	157,521	31 (NR)	Pfizer– BioNTech, Moderna, Oxford–AstraZeneca	Preterm birth, Very preterm birth, Stillbirth, Small for gestational age, Low Apgar score, Neonatal care admission	Good
Goldshtein [26]/2022	Israel	JAMA Pediatrics	Prospective cohort	March-September 2021	24,288	N.A	Pfizer– BioNTech	Preterm births, SGA, Inpatient hospitalizations, Recorded congenital anomalies, Jaundice requiring phototherapy, All cause death over the study period	Good
Fell [27]/2022	Canada	JAMA	Population-based retrospective cohort	December2020—September 2021	97,590	N.A	Pfizer– BioNTech, Moderna, Oxford–AstraZeneca	Postpartum hemorrhage, Chorioamnionitis, Cesarean delivery, Emergency cesarean delivery, Neonatal ICU (NICU) admission, Low newborn 5-min Apgar score	Good
Dick [28]/2022	Israel	BMC Pregnancy and Childbirth	Retrospective cohort	December2020—July 2021	5618	Median (IQR) Vaccinated (*n* = 2305): 30(26–34) Unvaccinated (*n* = 3313): 30(26–34)	Pfizer– BioNTech, Moderna	Preterm birth, Small for gestational age, Gestational diabetes and hypertensive disorders of pregnancy, Gestational age at delivery, Birthweight, Stillbirth, Mode of delivery, Postpartum hemorrhage, 5-min Apgar score, Umbilical artery pH and base excess	Good
Citu [29]/2022	Romania	Viruses	Prospective cohort	May—December 2021	702	Vaccinated (*n* = 173): 29.8 ± 6.1 Unvaccinated (*n* = 529): 31.2 ± 6.6	Pfizer– BioNTech, Johnson & Johnson	Gestational diabetes mellitus, Gestational hypertension, Oligohydramnios, Polyhydramnios, Placental abruption, Assisted birth, Cesarean delivery, Preterm delivery, Postpartum hemorrhage, Hospital stays, days, APGAR score < 7 at 5 min, Abnormal fetal monitoring, Meconium aspiration, Small for gestational age, Weight, Fever, ARDS	Fair
Blakeway[30]/2022	United Kingdom	American journal of obstetrics and gynecology	Retrospective cohort	March—July 2021	1328	At least 1 dose during pregnancy (*n* = 140): 35.0 (31.7–37.0) Did not receive a vaccine during pregnancy (*n* = 1188): 33.0 (30.0–36.0)	Pfizer– BioNTech, Moderna, Oxford–AstraZeneca	Stillbirth, Neonatal death, Fetal abnormalities, Preterm birth before 37 weeks’ gestation, GA at birth in weeks, Intrapartum complications (pyrexia, suspected chorioamnionitis, placental abruption, and postpartum hemorrhage), Birthweight z score, Mode of birth (cesarean delivery, instrumental delivery, or unassisted vaginal delivery), Maternal high-dependency unit or intensive care unit (ICU) admission, Neonatal ICU admission	Good
Beharier [6]/2021	Israel	The Journal of clinical investigation	Prospective cohort	April 2020—March 2021	1094	Control group (*n* = 66): 31.6 ± 5.8 Past SARS-CoV-2 group (*n* = 74):28.8 ± 5.8 Vaccinated group (*n* = 92):31.7 ± 5.8	Pfizer– BioNTech	Gestational age, Preterm delivery (< 37), Birthweight, NICU, Maternal and cord blood serological IgG response to S1, S2, RBD, and N antigens	Good

### Adverse neonatal outcomes (ANO)

**Fig. 2:**
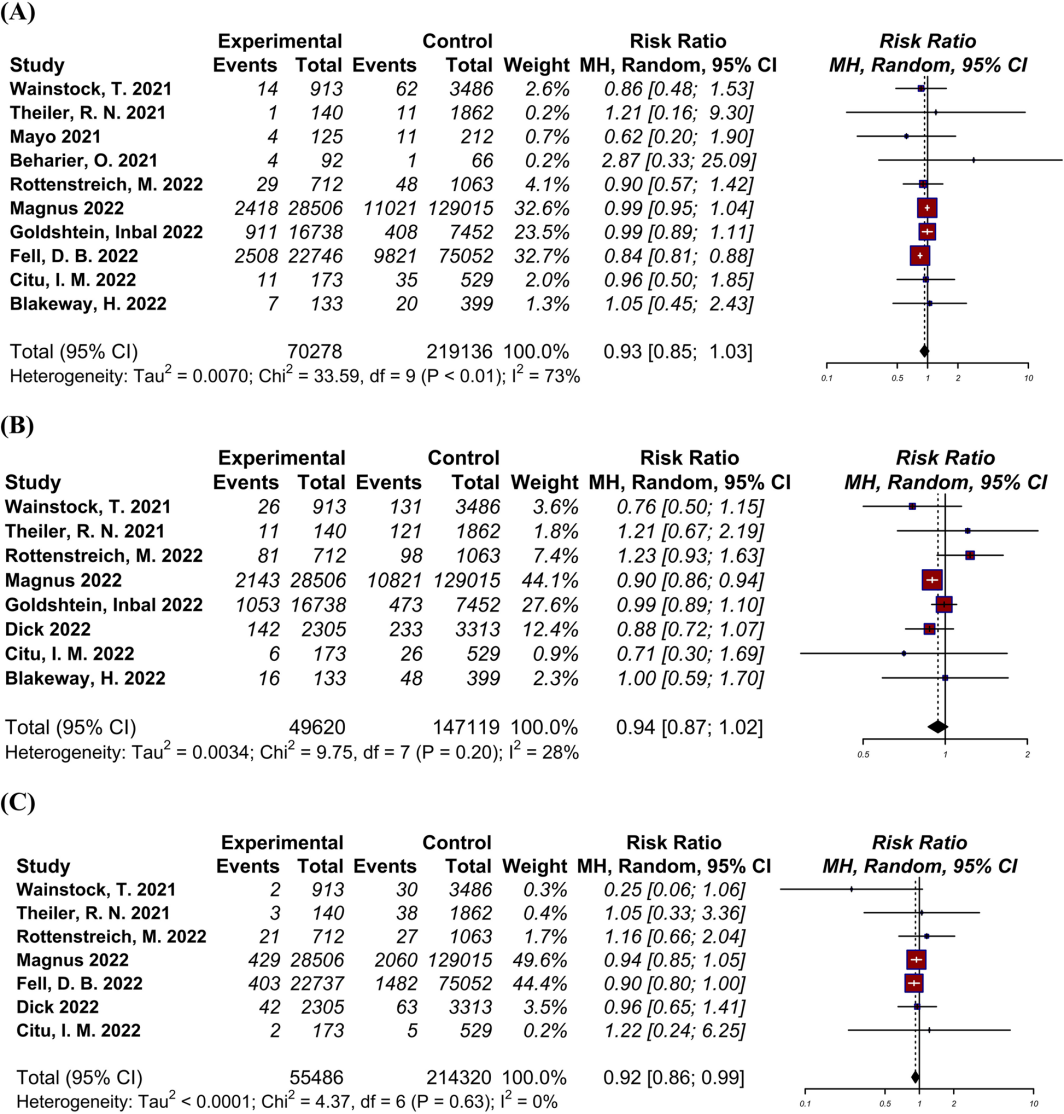
Forest plots showing the results of meta-analyses for neonatal outcomes. **A** Adverse neonatal outcome, **B** Small for gestational age, **C** 5-min Apgar score ≤ 7

A total of ten studies [4, 6, 22–27, 29, 30], including 289,414 pregnant women, reported the rate of NICU admission/newborn respiratory complications among neonates of vaccinated and unvaccinated pregnant mothers (Fig. 2-A). Log RR was -0.07 (95% CI: -0.17 to 0.03) indicating moderate amount of heterogeneity (I2 = 67.7%, Tau = 0.01, *p* < 0.01). Comparison of adverse neonatal outcomes showed no significant difference between the vaccinated and unvaccinated groups.

### Small for gestational age (SGA)

A total of eight studies [4, 22–24, 26, 28–30], including a total of 196,739 pregnant women, evaluated SGA among neonates of vaccinated and unvaccinated pregnant mothers and their neonates (Fig. 2-B). Log RR was -0.06 (95% CI: -0.14 to 0.02) indicating low amount of heterogeneity (I2 = 30.9%, Tau = 0.00, *p* = 0.20). There was no significant difference in SGA between the vaccinated and unvaccinated groups.

### 5-min Apgar score ≤ 7

A total of seven studies [22–24, 27–30], including 269,806 pregnant women, evaluated the Apgar score among neonates of vaccinated and unvaccinated groups (Fig. 2-C). Log RR was -0.08 (95% CI: -0.15 to -0.00) indicating low amount of heterogeneity (I2 = 0.0%, Tau = 0.00, *p* = 0.64). The rate of neonates with 5-min Apgar score ≤ 7 was significantly lower in the vaccinated group A significantly lower rate of neonates with 5-min Apgar score ≤ 7 was observed in the vaccinated group(*p* = 0.037).

### Caesarean delivery

**Fig. 3:**
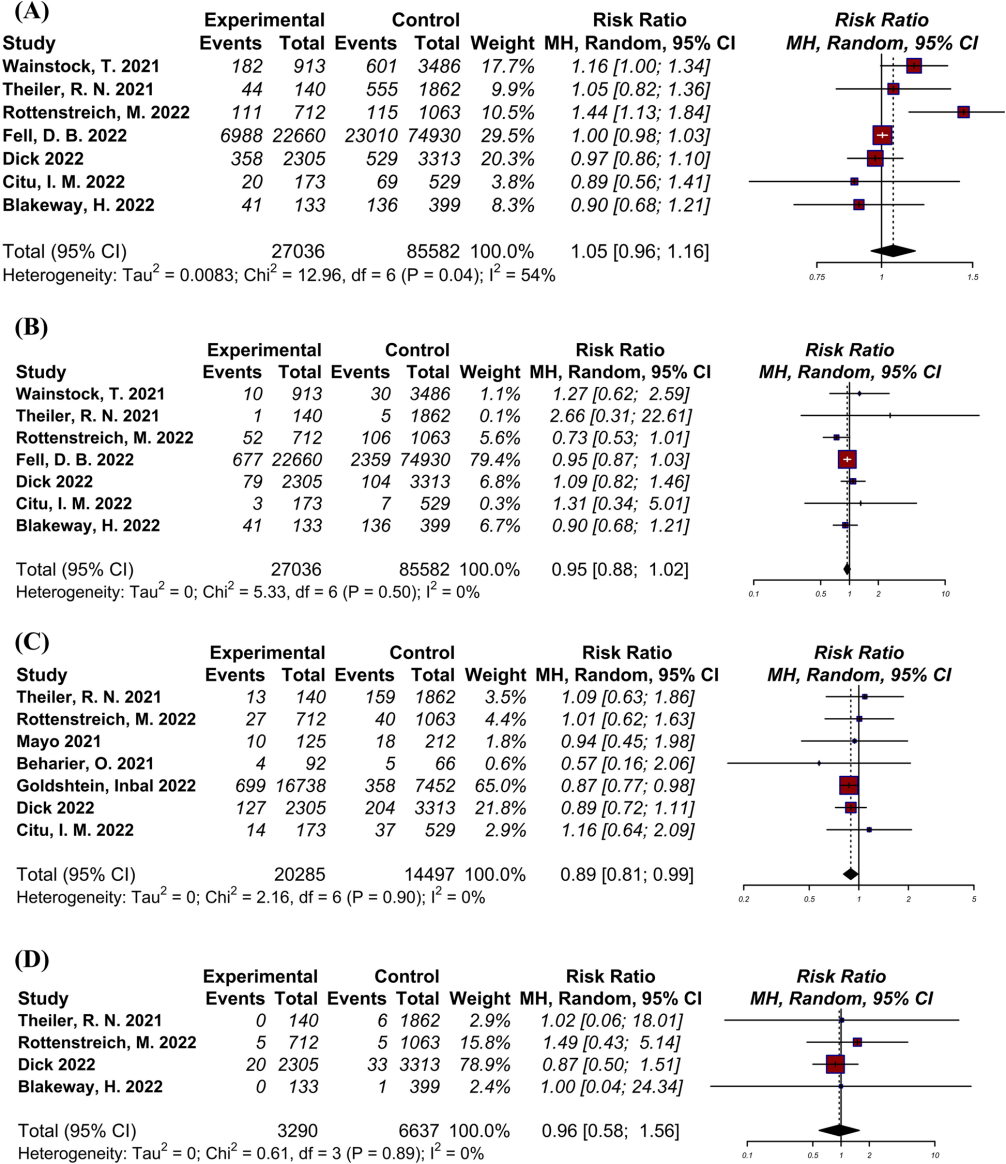
Forest plots showing the results of meta-analyses for pregnancy outcomes. **A** Caesarean delivery, **B** Postpartum hemorrhage, **C** Preterm birth, **D** Stillbirth

A total of seven studies [22–24, 27–30], including 112,618 pregnant women, compared the rate of caesarean delivery between vaccinated and unvaccinated groups (Fig. 3-A). Log RR was 0.05 (95% CI: -0.05 to -0.15) indicating moderate amount of heterogeneity (I2 = 61.9%, Tau = 0.01, *p* = 0.04). No significant difference between groups was observed.

### Postpartum hemorrhage (PPH)

A total of seven studies [22–24, 27–30], including 112,618 pregnant women, evaluated the rate of PPH (Fig. 3-B). Log RR was -0.05 (95% CI: -0.13 to 0.02) indicating low amount of heterogeneity (I2 = 0.0%, Tau = 0.00, *p* = 0.50). There was no significant difference between groups.

### Preterm birth

A total of seven studies [6, 23–26, 28, 29], including 34,782 pregnant women, reported the rate of preterm neonates in vaccinated and unvaccinated groups (Fig. 3-C). Log RR was -0.11 (95% CI: -0.21 to -0.01) indicating low amount of heterogeneity (I^2^ = 0.0%, Tau = 0.00, *p* = 0.90). The reate of preterm birth was significantly lower in vaccinated group (*p* = 0.0282).

### Stillbirth

A total of four studies [23, 24, 28, 30], including 9927 pregnant women, reported the rate of stillbirth vaccinated and unvaccinated groups (Fig. 3-D). Log RR was -0.05 (95% CI: -0.54 to 0.45) indicating low amount of heterogeneity (I^2^ = 0.0%, Tau = 0.00, *p* = 0.89). Our analysis showed no significant difference between groups.

### Sensitivity analysis and publication bias

Based on a leave-one out method, we evaluate the effect of removing individual study on the pooled results. We evaluated the effect of individual studies on the pooled results by employing a leave-one out method. The results of the sensitivity analysis for ANO, caesarean delivery, PPH, and preterm birth showed the pooled effect size was remained non-significant. The pooled results for three outcomes differed after omitting an individual study: 1) When excluding Rottenstreich et al. the risk of SGA for vaccinated group was significantly lower compring the unvaccinated (*p* = 0.01); 2) no significant difference was shown between the two groups after excluding Magnus et al. (*p* = 0.07) and Fell (*p* = 0.26) et al., respectively, in terms of the risk of 5-min Apgar < 7; and 3.Exclusion of Goldshtein (*p* = 0.48) and Dick (*p* = 0.51) et al.did not cause any significant difference between the two groups in terms of the risk of preterm birth (Table 2).

**Table 2:** Results of the sensitivity analyses

	**Author**	**Estimate**	**SE**	**p-val**	**Sig**
**Adverse neonatal outcome**					
1	Wainstock, T	-0.067	0.051	0.190	ns
2	Theiler, R. N	-0.070	0.049	0.160	ns
3	Mayo	-0.066	0.050	0.185	ns
4	Beharier, O	-0.071	0.049	0.149	ns
5	Rottenstreich, M	-0.067	0.052	0.194	ns
6	Magnus	-0.099	0.060	0.102	ns
7	Goldshtein, Inbal	-0.088	0.061	0.147	ns
8	Fell, D. B	-0.008	0.020	0.672	ns
9	Citu, I. M	-0.069	0.050	0.169	ns
10	Blakeway, H	-0.070	0.050	0.159	ns
**small for gestational age**					
1	Wainstock, T	-0.050	0.045	0.271	ns
2	Theiler, R. N	-0.066	0.041	0.110	ns
3	Rottenstreich, M	-0.085	0.034	0.012	*
4	Magnus	-0.020	0.043	0.635	ns
5	Goldshtein, Inbal	-0.081	0.049	0.101	ns
6	Dick	-0.043	0.053	0.423	ns
7	Citu, I. M	-0.058	0.043	0.178	ns
8	Blakeway, H	-0.062	0.043	0.150	ns
**5-min Apgar < 7**					
1	Wainstock, T	-0.074	0.037	0.047	*
2	Theiler, R. N	-0.078	0.037	0.037	*
3	Rottenstreich, M	-0.081	0.037	0.031	*
4	Magnus	-0.095	0.052	0.070	ns
5	Fell, D. B	-0.056	0.050	0.258	ns
6	Dick	-0.078	0.038	0.038	*
7	Citu, I. M	-0.078	0.037	0.037	*
**Caesarean delivery**					
1	Wainstock, T	0.033	0.056	0.562	ns
2	Theiler, R. N	0.055	0.060	0.362	ns
3	Rottenstreich, M	0.006	0.011	0.604	ns
4	Fell, D. B	0.072	0.069	0.295	ns
5	Dick	0.075	0.062	0.231	ns
6	Citu, I. M	0.060	0.053	0.256	ns
7	Blakeway, H	0.068	0.055	0.218	ns
**Postpartum hemorrhage**					
1	Wainstock, T	-0.058	0.038	0.130	ns
2	Theiler, R. N	-0.056	0.038	0.142	ns
3	Rottenstreich, M	-0.040	0.039	0.311	ns
4	Fell, D. B	-0.057	0.103	0.582	ns
5	Dick	-0.065	0.040	0.099	ns
6	Citu, I. M	-0.056	0.038	0.144	ns
7	Blakeway, H	-0.052	0.040	0.192	ns
**Preterm birth**					
1	Theiler, R. N	-0.119	0.052	0.022	*
2	Rottenstreich, M	-0.118	0.052	0.024	*
3	Mayo	-0.113	0.052	0.028	*
4	Beharier, O	-0.110	0.051	0.033	*
5	Goldshtein, Inbal	-0.061	0.087	0.482	ns
6	Dick	-0.113	0.058	0.051	ns
7	Citu, I. M	-0.120	0.052	0.021	*
**Stillbirth**					
1	Theiler, R. N	-0.047	0.254	0.853	ns
2	Rottenstreich, M	-0.129	0.273	0.637	ns
3	Dick	0.302	0.546	0.580	ns
4	Blakeway, H	-0.046	0.254	0.855	ns

Asterisks indicate studies that have a significant effect on the pooled estimate after omission

*SE* Standard error

Regarding publication bias, there was only one outcome with at least ten studies to evaluate ANO. A funnel plot of the ANO estimates is shown in Supplementary Fig. 1. The regression test demonstrated no sign of funnel plot asymmetry (*p* = 0.8377).

### Quality assessment and certainty of evidence

According to NHLBI checklist, most of the included studies were juded to be of Good/Fair quality. The majority of included studies did not provide details regarding the blinding of outcome assessors to the participants' exposure status or assessing the exposure in more than one study (Further details are available in the Supplementary Table 2). Moreover, the certainty of evidence for study outcomes are available in Table 3.

**Table 3:** Assessment of the quality of evidence based on GRADE approach

**Patient or population: Outcomes of pregnant women received COVID-19 vaccine compared with unvaccinated group**		
**Outcomes**	**No of participants** **(studies)**	**Certainty of the evidence** **(GRADE)**
**Adverse neonatal outcome**	289,414 (10 observational studies)	⨁⨁⨁◯ Moderate
**Small for gestational age**	196,739 (8 observational studies)	⨁⨁⨁◯ Moderate
**5-min Apgar < 7**	269,806 (7 observational studies)	⨁◯◯◯ Very low^a,b^
**Cesarean delivery**	112,618 (7 observational studies)	⨁⨁⨁◯ Moderate
**Post-partum hemorrhage**	112,618 (7 observational studies)	⨁⨁⨁◯ Moderate
**Preterm birth**	34,782 (7 observational studies)	⨁⨁◯◯ Low ^b^
**Stillbirth**	9927 (4 observational studies)	⨁⨁◯◯ Low^c^

GRADE Working Group grades of evidence. High certainty: We are very confident that the true effect lies close to that of the estimate of the effect. Moderate certainty: We are moderately confident in the effect estimate: The true effect is likely to be close to the estimate of the effect, but there is a possibility that it is substantially different. Low certainty: Our confidence in the effect estimate is limited: The true effect may be substantially different from the estimate of the effect. Very low certainty: We have very little confidence in the effect estimate: The true effect is likely to be substantially different from the estimate of effect

^a^ Imprecision

^b^ Inconsistency between the pooled result and the result of most of the included studies

^c^ Small sample size

## Discussion

It is well-known from the experience of influenza and pertussis that prevention of infections through vaccination is effective in decreasing both maternal and prenatal undesirable outcomes [31]. The current systematic review and meta-analysis was conducted with the aim of evaluating the effect of COVID-19 vaccination during pregnancy on neonatal and pregnancy outcomes. Our review included 11 observational studies with 756,098 participants. Several outcomes were assessed, including postpartum hemorrhage, preterm birth, stillbirth, caesarean delivery, and a low 5-min Apgar score (< 7). No significant differences were found regarding adverse neonatal outcomes, small for gestational age, caesarean delivery, postpartum hemorrhage, and stillbirth. However, our analyses showed that COVID-19 vaccination during pregnancy significantly decreases the incidence of preterm birth and low 5-min Apgar score [7] compared to the unvaccinated group with low certainty of evidence. Further research, including studies with larger sample sizes from different countries and sociodemographic diversity, are required to confirm our findings.

Current evidence shows that SARS-Cov-2 infection during pregnancy is associated with a higher risk of developing COVID-19 complications. The risk of maternal hospitalization, ICU admission, need for mechanical ventilation, and even death is higher among pregnant patients compared to their non-pregnant counterparts. Furthermore, they showed significantly higher rates of adverse pregnancy outcomes such as preterm birth and stillbirth [5, 32]. Therefore, it is crucial for this group to get vaccinated to prevent possible complications caused by the disease affecting both the mother and the fetus. Early vaccine trials only included non-pregnant women. On the other hand, due to physical alterations of the human body during pregnancy, special attention should be given to the safety measures of the vaccines for pregnant women. As pregnant women are more cautious about receiving a new vaccine, they are reluctant to receive vaccines. Therefore, they should be provided with adequate information available regarding this issue to enable them to make informed decisions regarding vaccination [33].

There are studies suggesting that maternal vaccination with the proper transfer of neutralizing antibodies through the placenta could potentially induce offspring immunity. This is particularly beneficial since neonates and infants are more susceptible to severe illness caused by COVID-19 compared to their older pediatric counterparts, especially, when there is no current approved vaccine used in children younger than two years old [34, 35]. Beharier et al. showed that antenatal BNT162b2 mRNA vaccination induces a robust maternal immune response that is followed by an effective transfer of protective antibodies and a rise in their amount in the fetal circulation, emphasizing the importance of vaccination against COVID-19 during pregnancy [6].

Studies included in our review reported that there is no significant association between SARS-CoV-2 vaccination during pregnancy and an increased risk of adverse pregnancy outcomes. Rottenstreich et al. stated that based on their adjusted multivariable logistic regression analysis, the rate of composite adverse neonatal outcomes was lower among the vaccinated group. However, none of the individual neonatal outcomes were different between the two groups [36]. Magnus et al. showed that the risk of neonatal care admission and low Apgar scores was modestly decreased following vaccination during the third trimester [4]. In Dick et al.’s study, an increased rate of preterm birth was observed among pregnant women vaccinated during the second trimester in comparison with unvaccinated pregnant women [28]. Additionally, Goldshtein et al. observed that the rate of congenital malformation in the vaccinated group was not higher than the unvaccinated group and was similar to prepandemic reports [26].

There are some sociodemographic factors that contribute to a disparity between populations in terms of vaccination rates. Studies reported that older age, higher level of maternal education, higher socioeconomic position, conceiving following fertility treatment, having sufficient prenatal care, and lower gravidity are associated with increased rates of vaccination [22, 23].

The current findings should give people and clinicians confidence that vaccination against COVID-19 protects individuals from maternal SARS-CoV-2 infection and is not associated with adverse pregnancy and neonatal outcomes. Efforts should be made to improve awareness of vaccine safety among pregnant women and health providers and to address the issue of vaccine hesitancy. There are some strengths in our study. As far as we are aware, the current study is the most comprehensive systematic review and meta-analysis conducted to evaluate the association of COVID-19 vaccination with pregnancy outcomes. Ma et al. conducted a systematic review and meta-analysis on this subject, including six observational studies [37]. De Rose et al.’s systematic review summarized the current knowledge about pregnancy outcomes related to vaccination during pregnancy and breastfeeding [38]. The findings of these studies are in line with ours. However, our review is more comprehensive when considering both the number of included studies and whether a meta-analysis was performed. We performed a thorough database search to obtain the most comprehensive set of underlying studies and achieve accurate results. For three of the outcomes, we included seven studies in the meta-analysis, with the rest including six and four studies. Most of the included studies had adjusted for confounding variables.Our study has limitations. Since the majority of the analyzed studies were cohorts, they might be potentially biased due to their retrospective design. Most of our data were extracted from observational studies of high-income countries, limiting us regarding the diversity of participants in terms of sociodemographic characteristics. Furthermore, vaccines used in the studies were primarily mRNA vaccines, and little data was available regarding other types of vaccines approved by the WHO and used worldwide, such as Sinopharm, Sinovac, COVAXIN, and Nuvaxoid. Therefore, further research is required to determine the safety of administering these vaccines during pregnancy. Most vaccines were administered in the second or third trimester of pregnancy. Even though few studies have examined the safety of vaccines during the first trimester, the authors call for more data on the precise time of vaccine administration and its safety to inform maternal, pregnancy, and infant outcomes.

## Conclusion

Our analyses show that vaccination against SARS-CoV-2 during pregnancy is not associated with a higher risk of adverse pregnancy and neonatal outcomes. Further research, including studies with larger sample sizes, more diverse populations, different types of vaccines, and variable timings for the administration of vaccines, is required to reach a solid conclusion regarding this issue.

## Supplementary Information

**Additional file 1: Supplementary Table 1.** Databases searched and search strategies employed. **Supplementary Table 2.** NIH Quality Assessment Checklist. **Supplementary Figure 1.** Funnel Plot of Studies Included in the Meta-analysis of adverse neonatal outcomes (ANO).

## Data Availability

Data sharing is available by contacting corresponding author.

## Abbreviations

SARS-CoV-2: Severe acute respiratory syndrome coronavirus 2
COVID-19: Coronavirus disease 2019
WHO: World Health Organization
NHLBI: National Heart Lung and Blood Institute
NICU: Neonatal intensive care unit
ANO: Adverse neonatal outcomes
SGA: Small for gestational age
PPH: Postpartum hemorrhage
