# Supplementary material for: COVID-19 vaccination during pregnancy: a systematic review and meta-analysis
Source: BMC Pregnancy Childbirth. 2023 Jan 20;23:45. doi: 10.1186/s12884-023-05374-2 (PMC9853484; doi:10.1186/s12884-023-05374-2)
Supplement: Supplementary file 1 — Additional file 1: Supplementary Table 1. Databases searched and search strategies employed. Supplementary Table 2. NIH Quality Assessment Checklist. Supplementary Figure 1. Funnel Plot of Studies Included in the Meta-analysis of adverse neonatal outcomes (ANO). [file 12884_2023_5374_MOESM1_ESM.docx]

**Supplementary data**

**Supplementary Table 1: Databases searched and search strategies employed**

**Date: 4/7/2022- Total: 454, Pubmed:148, EMBASE: 147, WOS: 159, After dup: 282- CT.GOV:182**

| **Database** | **Search strategy** |
| --- | --- |
| PubMed | (((((((("SARS-CoV-2"[Mesh]) OR "COVID-19"[Mesh]) OR (COVID-19[Title/Abstract])) OR (Coronavirus[Title/Abstract])) OR (nCoV[Title/Abstract])) OR (SARS-Cov-2[Title/Abstract])) AND ((("Vaccines"[Mesh]) OR (vaccines[Title/Abstract])) OR (vaccin*[Title/Abstract]))) AND (((Pregnan*[Title/Abstract]) OR (prenatal[Title/Abstract])) OR (perinatal[Title/Abstract]))) AND ((((newborn[Title/Abstract]) OR (neonate[Title/Abstract])) OR (neonatal[Title/Abstract])) OR (infant[Title/Abstract])) |
| Web of Science | (COVID-19 (Topic) or Coronavirus (Topic) or nCoV (Topic) or SARS-Cov-2 (Topic)) AND (Vaccines (Topic) or Vaccine (Topic) or Vaccin* (Topic)) AND (pregnancy (Topic) or pregnant (Topic) or pregnan* (Topic) or prenatal (Topic) or perinatal (Topic)) AND (newborn (Topic) or neonate (Topic) or neonatal (Topic) or infant (Topic)) |
| EMBASE | ('coronavirus disease 2019':ab,ti OR 'covid 19':ab,ti OR coronavirus:ab,ti OR ncov:ab,ti OR 'sars cov 2':ab,ti) AND (vaccine:ab,ti OR vaccines:ab,ti OR vaccin*:ab,ti) AND (pregnancy:ab,ti OR pregnant:ab,ti OR pregnan*:ab,ti OR prenatal:ab,ti OR perinatal:ab,ti) AND (newborn:ab,ti OR neonate:ab,ti OR neonatal:ab,ti OR infant:ab,ti) |

**Supplementary Table 2: NIH Quality Assessment Checklist**

| **Study id** | **1.Clarity** | **2.Population** | **3.Participation rate (>50%)** | **4.simillar population/ inclusion and exclusion criteria** | **5.sample justification** | **6.exposure/outcome** | **7.timeframe** | **8.different levels of exposure** | **9.exposure measures** | **10.repeated exposure assessment** | **11.outcome measures** | **12.outcome blinded** | **13. follow-up rate (80%)** | **14.statistical adjusment of cofounding variables** | **Total score** | **Total assessment** |
| --- | --- | --- | --- | --- | --- | --- | --- | --- | --- | --- | --- | --- | --- | --- | --- | --- |
| Magnus/2022 | 1 | 1 | 1 | 1 | 0 | 1 | 1 | NA | 1 | NA | 1 | NA | 1 | 1 | 10 | GOOD |
| Goldshtein/2022 | 1 | 1 | 1 | 1 | 0 | 1 | 1 | NA | 1 | NA | 1 | NA | 1 | 0 | 9 | GOOD |
| Fell/2022 | 1 | 1 | 1 | 1 | 0 | 1 | 1 | NA | 1 | NA | 1 | NA | 1 | 1 | 10 | GOOD |
| Dick/2022 | 1 | 1 | 1 | 1 | 0 | 1 | 1 | NA | 1 | NA | 1 | NA | 1 | 1 | 10 | GOOD |
| Citu/2022 | 0 | 1 | NA | 1 | 0 | 1 | 1 | NA | 1 | NA | 0 | NA | 1 | 0 | 6 | FAIR |
| Blakeway/2021 | 1 | 1 | 0 | 1 | 0 | 1 | 1 | NA | 1 | NA | 1 | NA | 1 | 1 | 9 | GOOD |
| Beharier/2021 | 1 | 1 | 1 | 1 | 0 | 1 | 1 | NA | 1 | 0 | 1 | 1 | 1 | 1 | 11 | GOOD |
| Tamar wainstock/ 2021 | 1 | 1 | 1 | 1 | 1 | 1 | 1 | NA | 1 | 0 | 1 | NA | NA | 1 | 10 | GOOD |
| Theiler/2021 | 1 | 1 | 1 | 1 | 1 | 1 | 1 | 1 | 1 | 0 | 1 | NA | NA | 1 | 11 | GOOD |
| Rottenstreich/2022 | 1 | 1 | 1 | 1 | 1 | 1 | 1 | NA | 1 | 0 | 1 | NA | NA | 1 | 10 | GOOD |
| Mayo/2021 | 1 | 1 | 1 | 1 | 1 | 1 | 1 | 1 | 1 | 0 | 1 | NA | NA | 0 | 10 | GOOD |

**Supplementary Figure 1. Funnel Plot of Studies Included in the Meta-analysis of**
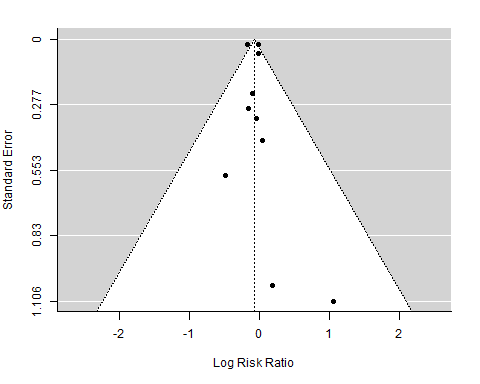
**adverse neonatal outcomes (ANO).**
